# Indirect overgrowth as a synthesis route for superior diamond nano sensors

**DOI:** 10.1038/s41598-020-79943-2

**Published:** 2020-12-29

**Authors:** Christoph Findler, Johannes Lang, Christian Osterkamp, Miloš Nesládek, Fedor Jelezko

**Affiliations:** 1 Institute for Quantum Optics and Center for Integrated Quantum Science and Technology (IQST), Ulm University, Albert-Einstein-Allee 11, 89081 Ulm, Germany; 2 Mercedes-Benz AG, RD/EB, HPC X461, 71059 Sindelfingen, Germany; 3 Institute for Materials Research (IMO), Hasselt University, Wetenschapspark 1, 3590 Diepenbeek, Belgium

**Keywords:** Materials science, Nanoscience and technology, Physics

## Abstract

The negatively charged nitrogen-vacancy ($\hbox {NV}^{-}$) center shows excellent spin properties and sensing capabilities on the nanoscale even at room temperature. Shallow implanted $\hbox {NV}^{-}$ centers can effectively be protected from surface noise by chemical vapor deposition (CVD) diamond overgrowth, i.e. burying them homogeneously deeper in the crystal. However, the origin of the substantial losses in $\hbox {NV}^{-}$ centers after overgrowth remains an open question. Here, we use shallow $\hbox {NV}^{-}$ centers to exclude surface etching and identify the passivation reaction of NV to NVH centers during the growth as the most likely reason. Indirect overgrowth featuring low energy (2.5–5 keV) nitrogen ion implantation and CVD diamond growth before the essential annealing step reduces this passivation phenomenon significantly. Furthermore, we find higher nitrogen doses to slow down the NV–NVH conversion kinetics, which gives insight into the sub-surface diffusion of hydrogen in diamond during growth. Finally, nano sensors fabricated by indirect overgrowth combine tremendously enhanced $T_2$ and $T_2^*$ times with an outstanding degree of depth-confinement which is not possible by implanting with higher energies alone. Our results improve the understanding of CVD diamond overgrowth and pave the way towards reliable and advanced engineering of shallow $\hbox {NV}^{-}$ centers for future quantum sensing devices.

## Introduction

Over the last two decades, quantum metrology with negatively charged nitrogen-vacancy ($\hbox {NV}^{-}$) centers in diamond has become increasingly important in the fields of information^1^, materials^2^ and life science^3^. The $\hbox {NV}^{-}$ center is sensitive to various physical quantities such as temperature^4–6^, strain^7^, electric^8^ and magnetic fields^9–12^. Optical polarization^13^ and read-out^14^ enable the coherent control of the electron spin ($S=1$) of the $\hbox {NV}^{-}$ center. Furthermore, it exhibits coherence times up to milliseconds at room temperature^15,16^ which makes the $\hbox {NV}^{-}$ center a promissing candidate for quantum sensing under ambient conditions^17^. Consisting of a substitutional nitrogen, a carbon-vacancy and an additional donor electron from the diamond lattice, the $\hbox {NV}^{-}$ center is a powerful nanoscale sensor^9,10,18–24^.

To achieve a high sensitivity to external spins the $\hbox {NV}^{-}$ center has to be positioned a few nanometers below the diamond’s surface^6,20,22,25–27^, since the measurable signal decays with the third power of the distance. These shallow $\hbox {NV}^{-}$ centers, however, suffer from decoherence^28,29^ and charge-instability^30^, probably due to (paramagnetic) defects at the surface. With increasing depth of the $\hbox {NV}^{-}$ centers this surface related influence is reduced, resulting in longer coherence times^28,29,31^. For magnetic field sensing applications this is beneficial since the smallest detectable field is inversely proportional to the square root of the coherence time $T_{2}$ or the dephasing time $T_{2}^*$ for ac- and dc-fields, respectively^11^. Moreover, long coherence times are favorable for applications involving quantum registers where the NV–NV dipole detection limit strongly depends on $T_{2}$^32^. In our opinion, this trade-off finds an optimum in the so-called intermediate-regime (depths of 10–30 nm) which is characterized by significantly extended spin lifetimes while still preserving sufficient sensitivity to external spins on the surface. For instance, such intermediate $\hbox {NV}^{-}$ centers could be employed to study viscous liquids in microfluidic devices^33^.

Finally, the tailored fabrication of $\hbox {NV}^{-}$ centers with a narrow depth distribution combined with the possibility of increasing their coherence times by slightly enlarging the average depth to the surface is therefore key for several applications mentioned above.

Low energy implantation of $\hbox {NV}^{-}$ centers and the subsequent overgrowth of a diamond layer by chemical vapor deposition (CVD) has been associated with a significant enhancement of the coherence time, as reported by Staudacher et al.^29^. By tuning the average depth of implanted $\hbox {NV}^{-}$ centers solely by the thickness of a diamond capping layer, both the ion straggle and the implantation damage can be minimized due to the possibility to choose generally lower implantation energies. But it has also been observed that implanted NV centers are either etched^29,34^ and/or passivated^34,35^ by the hydrogen plasma essential for CVD diamond overgrowth. The prevailing mechanism and the crucial process parameters promoting either the former or the latter one are still unclear, though.

A likely passivation mechanism is the creation of non-fluorescing NVH centers from NV centers and diffusing hydrogen atoms from the plasma^35^. For intrinsic diamond, it is believed that hydrogen diffuses up to 80 microns into the crystal and passivates NV centers^35^. The paramagnetic $\hbox {NVH}^{-}$ defect is very stable in terms of temperature^36^ and is likely to be found in CVD-diamond^37–39^. The conversion of NV into NVH centers, however, has not been proven, yet. Using electron-paramagnetic resonance (EPR) spectroscopy the ratio between $\hbox {NV}^{-}$ and $\hbox {NVH}^{-}$ can be determined for bulk samples but for shallow ion implantation where nitrogen is distributed only in the first ten nanometers below the diamond’s surface the actual sample volume remains too small for this method^39^. By contrast, the number of $\hbox {NV}^{-}$ centers can be determined optically even in thin layers using confocal microscopy^40^. This enables isolating the crucial process parameters to limit the losses in $\hbox {NV}^{-}$ centers and finally optimizing the implantation and overgrowth approach.

Here, we examine the overgrowth of implanted $^{15}\hbox {NV}^{-}$ centers as a way to precisely tailor the properties of spin qubits in diamond and finally propose a modified approach called indirect overgrowth which limits the passivation of $^{15}\hbox {NV}^{-}$ centers during the process. In contrast to the existing reports^29,34^, we track the $\hbox {NV}^{-}$ density, the depth and the spin properties of overgrown $\hbox {NV}^{-}$ centers for different capping layer thicknesses and implantation parameters. Furthermore, we also verify the success of the presented fabrication technique by measuring the isotope of the nitrogen nucleus associated with the $^{15}\hbox {NV}^{-}$ centers. Note that the nitrogen isotope $^{15}\hbox {N}$ accounts only for 0.4 % of all nitrogen in nature. This procedure is absolutely necessary to clearly demonstrate the stabilization and improvement effect of diamond overgrowth. Otherwise, there is a risk of confusion with native $^{14}\hbox {NV}^{-}$ centers lying possibly much deeper in the diamond crystal with intrinsically longer coherence times.

## Results and discussion

**Figure 1:**
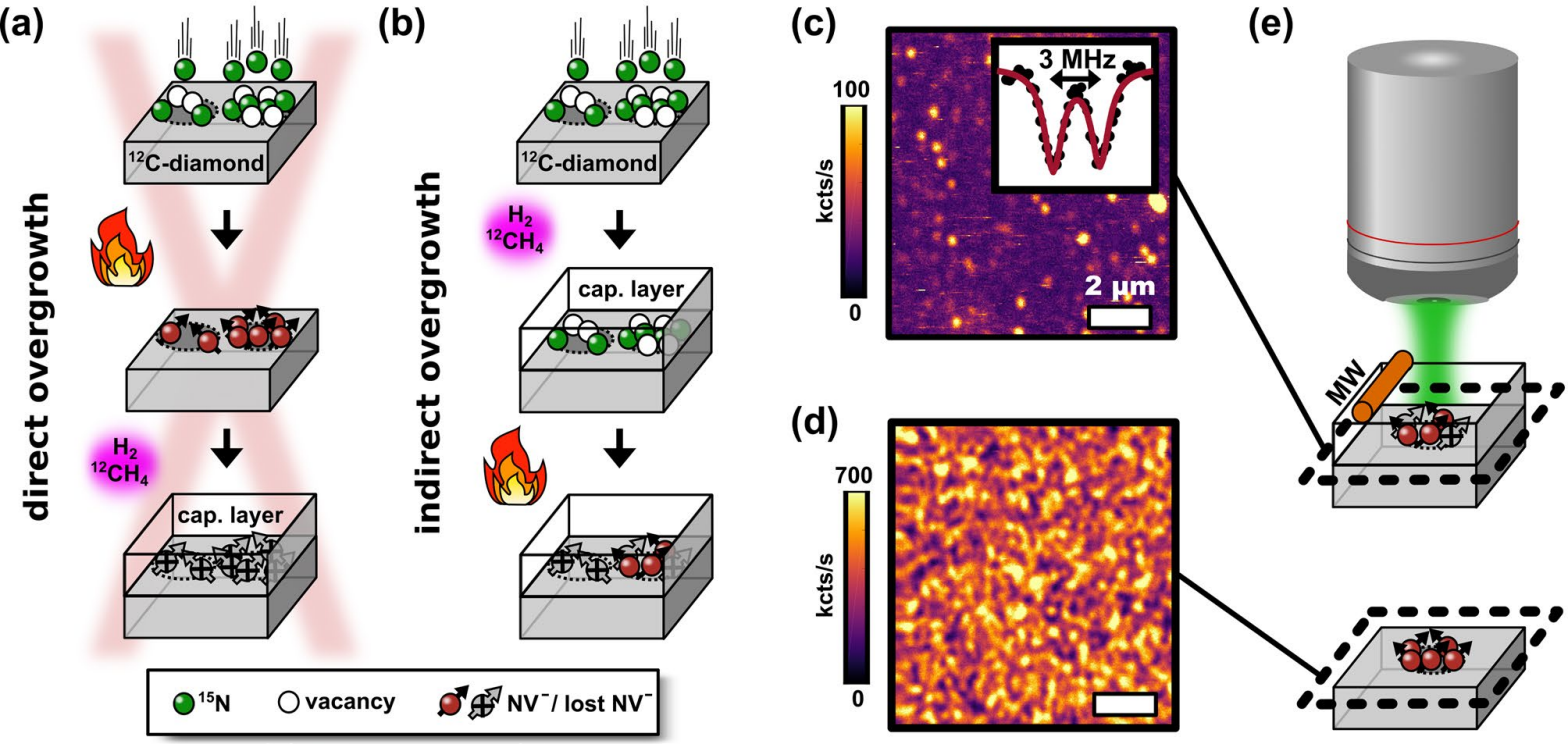
Combining ion implantation of $^{15}\hbox {N}^{+}$ and chemical vapor deposition (CVD) for the depth-controlled fabrication of $\hbox {NV}^{-}$ centers. (**a**) Direct overgrowth: $^{15}\hbox {N}^{+}$ nitrogen ion implantation into $99.999\,\%$ $^{12}\hbox {C}$ enriched diamond, ultra high vacuum (UHV) annealing at $1000\,^{\circ }\hbox {C}$ yielding $\hbox {NV}^{-}$ centers, and overgrowth of a nanometer-thick capping layer. The implantation spots are not visible anymore after the direct overgrowth of $\hbox {NV}^{-}$ centers. (**b**) Indirect overgrowth: analogous to (**a**) but overgrowing prior to annealing. The overgrowth of nitrogen and annealing at the end yields buried $\hbox {NV}^{-}$ centers for medium and high implantation doses ($10^{11}$ and $10^{12}\,^{15}\hbox {N}\,^{+}/\hbox {cm}^{2}$), whereas for the lowest dose ($10^{9}\,^{15}\hbox {N}\,^{+}/\hbox {cm}^{2}$) the implantation spots still vanish. (**c**) Confocal image showing the fluorescence of implanted single $\hbox {NV}^{-}$ centers (medium dose) after the indirect overgrowth with a capping layer of 13 nm thickness. Inset: a representative pulsed optically-detected-magnetic resonance measurement with two dips corresponding to the $^{15}\hbox {N}$-nuclear hyperfine interaction with the electron spin of the $\hbox {NV}^{-}$ center. (**d**) Reference image for (**c**) without overgrowth. (**e**) Scheme of the confocal measurements performed in (**b**) and (**c**) employing a 519 nm laser and coherent microwave control (MW) through a copper wire.

The sequence of $\hbox {NV}^{-}$ production via the so-called direct overgrowth procedure is depicted in Fig. 1(a): nitrogen ion implantation with subsequent annealing and overgrowth. The schematic in Fig. 1(b) shows the indirect overgrowth procedure where overgrowth is performed prior to annealing. The evaluation of both overgrowth methods is performed via confocal microscopy with green laser excitation (519 nm) through an scanning oil-immersion objective (Fig. 1(e)). In Fig. 1(c),(d) we show representative confocal images for an overgrown and a reference sample. Applying microwave pulses through a copper wire which serves as a microwave (MW) antenna (MW in Fig. 1(e)), we measure the nuclear hyperfine splitting of the $m_{s}=+1$ electron spin state using the pulsed optically detected magnetic resonance (pulsed ODMR) technique. The two Lorentzian-shaped dips with a frequency splitting of 3 MHz in the inset of Fig. 1(c) are associated with the $^{15}\hbox {N}$-nucleus ($I_{^{15}N}=1/2$) of the $\hbox {NV}^{-}$ center which confirms it to be implantation-induced^41^.

In this study, all samples are implanted with $^{15}\hbox {N}$ nitrogen ions at energies of 2.5 and 5 keV, respectively. The ion doses $10^{9}$, $10^{11}$, and $10^{12}$ $^{15}\hbox {N}^{+}/\hbox {cm}^{2}$ are in the following labeled as low, medium, and high dose, respectively. After performing the direct overgrowth (Fig. 1(a)) at $900\,^{\circ }\hbox {C}$ for one hour the implantation spots are not visible anymore. Under our growth conditions (cf. “Methods” section) most of the $\hbox {NV}^{-}$ centers seem therefore to get etched and/or passivated during the CVD process^29,34^. Hence, we conclude that the direct overgrowth is not successful in our case.

Assuming that nitrogen atoms do not react with hydrogen, we use the indirect overgrowth method, where implanted nitrogen is overgrown and finally converted into $\hbox {NV}^{-}$ centers by annealing (Fig. 1(b)). As a result, we observe $^{15}\hbox {NV}^{-}$ centers for the medium (Fig. 1(c)) and the high dose (not shown) even after overgrowing for two hours. At the growth temperature of $900\,^{\circ }\hbox {C}$, a part of the implanted nitrogen is probably already converted into $\hbox {NV}^{-}$ centers which get directly lost during overgrowth, as observed for the direct overgrowth. This circumstance is as well illustrated in Fig. 1(a). As a consequence, we still lose NV centers through the indirect overgrowth but it seems that we reduce the losses as long as the implanted nitrogen is not converted into NV centers. The fact that the state of the nitrogen (implanted nitrogen or NV) matters for the success of the overgrowth step might be an indicator for the passivation of NV centers.

In the following, only $\hbox {NV}^{-}$ centers originating from implantation with the medium dose are presented. Since we obtain mainly single $\hbox {NV}^{-}$ centers after overgrowth and annealing, we use a sample implanted with the low dose as a reference. The implantation dose has no influence on the resulting depth distribution of the $\hbox {NV}^{-}$ centers but alters their density.

**Figure 2:**
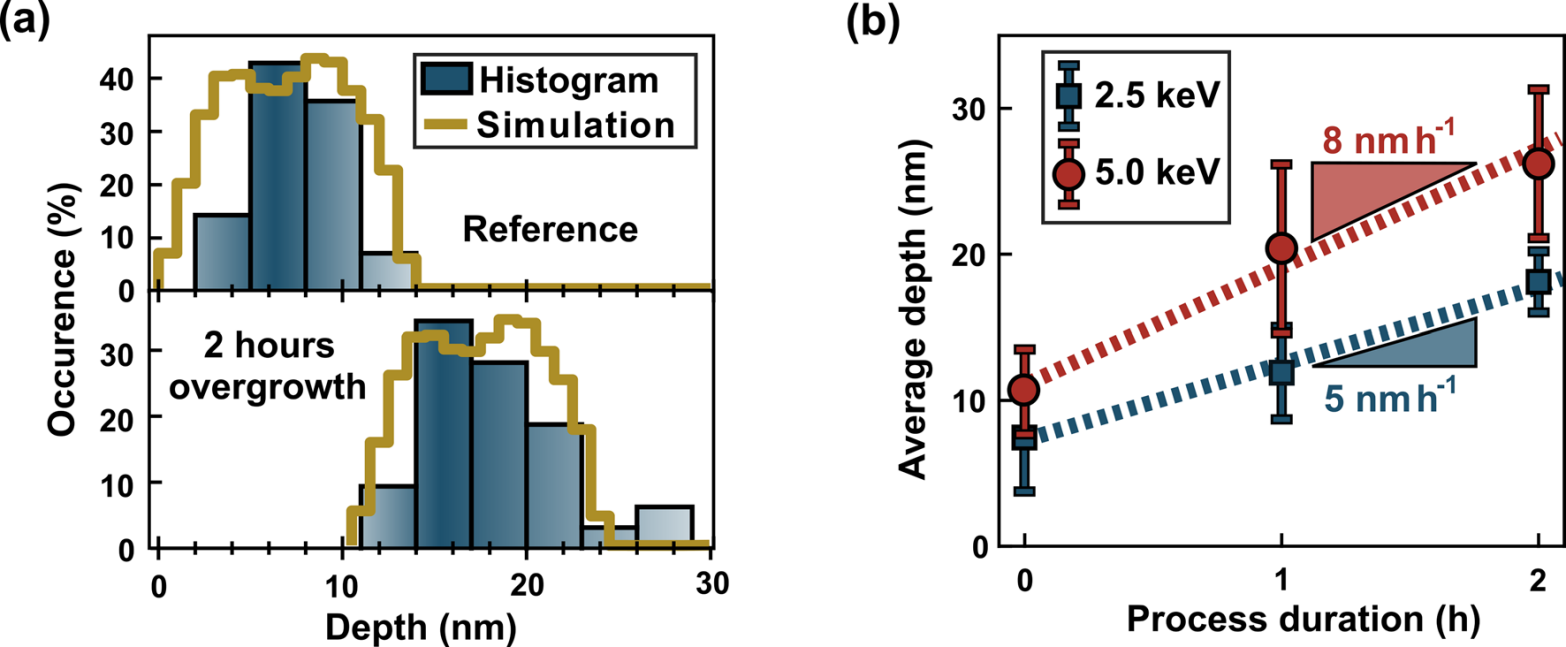
The change in depth of the fabricated $\hbox {NV}^{-}$ centers as a result of CVD-diamond overgrowth. (**a**) Histograms showing the depth distribution of single $\hbox {NV}^{-}$ centers in the reference sample implanted with the low dose (2.5 keV, $10^{9}\,^{15}\hbox {N}^{+}/\hbox {cm}^{2}$) and a second sample with the medium dose (2.5 keV, $10^{11}\,^{15}\hbox {N}^{+}/\hbox {cm}^{2}$) after indirect overgrowth for 2 h. The depth is calculated from sensing the NMR signal of the $^{1}\hbox {H}$-spins in the immersion oil on top of the diamond sample^42^. The simulated depth distribution from C-TRIM^43^ is shifted by 10 nm for the overgrown sample. (**b**) The evolution of the average depth of the $\hbox {NV}^{-}$ centers with increasing overgrowth time for implantation energies of 2.5 keV and 5 keV (medium dose). Zero hours corresponds to the reference sample in (**a**) and the error bars represent the standard deviation. The growth rates are extracted from a linear fit.

To study the impact of etching and to verify the success of the overgrowth step, we compare the depth distribution of $\hbox {NV}^{-}$ centers implanted with 2.5 keV after the indirect overgrowth for two hours with the reference sample. Corresponding histograms are shown in Fig. 2(a), where the depths of various $\hbox {NV}^{-}$ centers are determined by analyzing the nuclear magnetic resonance (NMR) signal of the $^{1}\hbox {H}$-spins in the immersion oil by using the electron spin of the $\hbox {NV}^{-}$ center as a sensor^22,42^. The measured depths for the reference sample agree with the theoretical distribution from C-TRIM simulations^43,44^. After the overgrowth the measured depths in Fig. 2(a) shift to deeper values whereas the overall shape of the distribution remains. Note that the, by 10 nm shifted, theoretical curve received from C-TRIM matches also nicely the depth distribution of the overgrown $\hbox {NV}^{-}$ centers.

Since the depth distribution itself is not altered by the indirect overgrowth we conclude that implantation-induced $\hbox {NV}^{-}$ centers are buried below a thin capping-layer of diamond. Furthermore, etching of the first few nanometers of diamond can be ruled out. Hydrogen-induced etching would remove the most shallow $\hbox {NV}^{-}$ centers first, causing a distinct narrowing of the depth distribution from the lower depth values’ side in Figure 2(a). As this effect cannot be observed, the loss of $\hbox {NV}^{-}$ centers during overgrowth must have another cause, promoting the hypothesis of NV passivation by hydrogen. The passivation and the influence of the implantation parameters are discussed later in this Article.

In Fig. 2(b) we plot the average depth of the $\hbox {NV}^{-}$ centers implanted with 2.5 keV and 5 keV against the duration of the overgrowth. The error bars correspond to the standard deviation ($1\upsigma$) of the individual depth distributions. The first result which can be extracted from the plot is that the average depth increases linearly with the overgrowth time. The slopes in Fig. 2(b) corresponds to the $\hbox {NV}^{-}$-calibrated diamond growth rate. For 2.5 keV we find $5\,\hbox {nm}\,\hbox {h}^{-1}$ whereas for 5 keV the slope is slightly steeper resulting in a higher rate of $8\,\hbox {nm}\,\hbox {h}^{-1}$. The deviation between the growth rates lies within the range of the error bars of Fig. 2(b). For further calculations we use the averaged growth rate of $6.5\,\hbox {nm}\,\hbox {h}^{-1}$. The calibration of the growth rate $\hbox {NV}^{-}$ centers as the probe is however restricted to sufficient coupling between the $\hbox {NV}^{-}$ center and the $^{1}\hbox {H}$-spins and is, therefore, only applicable to layer thicknesses of tens of nanometers, provided $T_{2}$ is long enough^42^.

So far we have shown that we are able to tune the average depth of implanted and overgrown $\hbox {NV}^{-}$ centers precisely and reproducibly on the nanometer scale. The advantage of implantation and subsequent overgrowth is the fact that we do not control the depth of the final $\hbox {NV}^{-}$ centers by increasing the implantation energy but through adjusting the duration of the overgrowth.

A higher kinetic energy of the nitrogen ions would result also in deeper $\hbox {NV}^{-}$ centers but at the cost of a broader depth distribution. Furthermore, the higher energy transfer would cause more damage and, therefore, more decoherence sources. In contrast to pure implantation, the overgrowth increases the distance of $\hbox {NV}^{-}$ centers to the surface without affecting the relative distribution of the $\hbox {NV}^{-}$ centers leading to the uniform shift in Fig. 2(a).

Additionally, decreasing the implantation energy reduces not only the ion straggle (error bars in Fig. 2(b)) but also the amount of vacancy related defects created upon implantation. Paramagnetic defects which do not anneal out at around $1000\,^{\circ }\hbox {C}$, for example divacancies (R4/W6 center) or more complex vacancy clusters or chains (R5, O1, R6 centers), can cause random magnetic field fluctuations limiting the spin lifetime of $\hbox {NV}^{-}$ centers^45^. Therefore, implantation and subsequent overgrowth is able to yield intermediate-depth $\hbox {NV}^{-}$ centers while avoiding unnecessary implantation damage. The overgrowth directly after ion implantation improved for example also the properties of tin-vacancy centers in diamond, as the implantation energy could be reduced^46^.

Defects at the growth interface, however, might also compromise the spin properties^47,48^ and, therefore, we plot $T_{2}$ (Hahn-echo) as a function of depth for several single $\hbox {NV}^{-}$ centers from the reference and two samples overgrown for one and two hours, respectively. Based on the determined average growth rate (Fig. 2(b)), the overgrown layers are expected to have a thickness of 6.5 nm and 13 nm.

**Figure 3:**
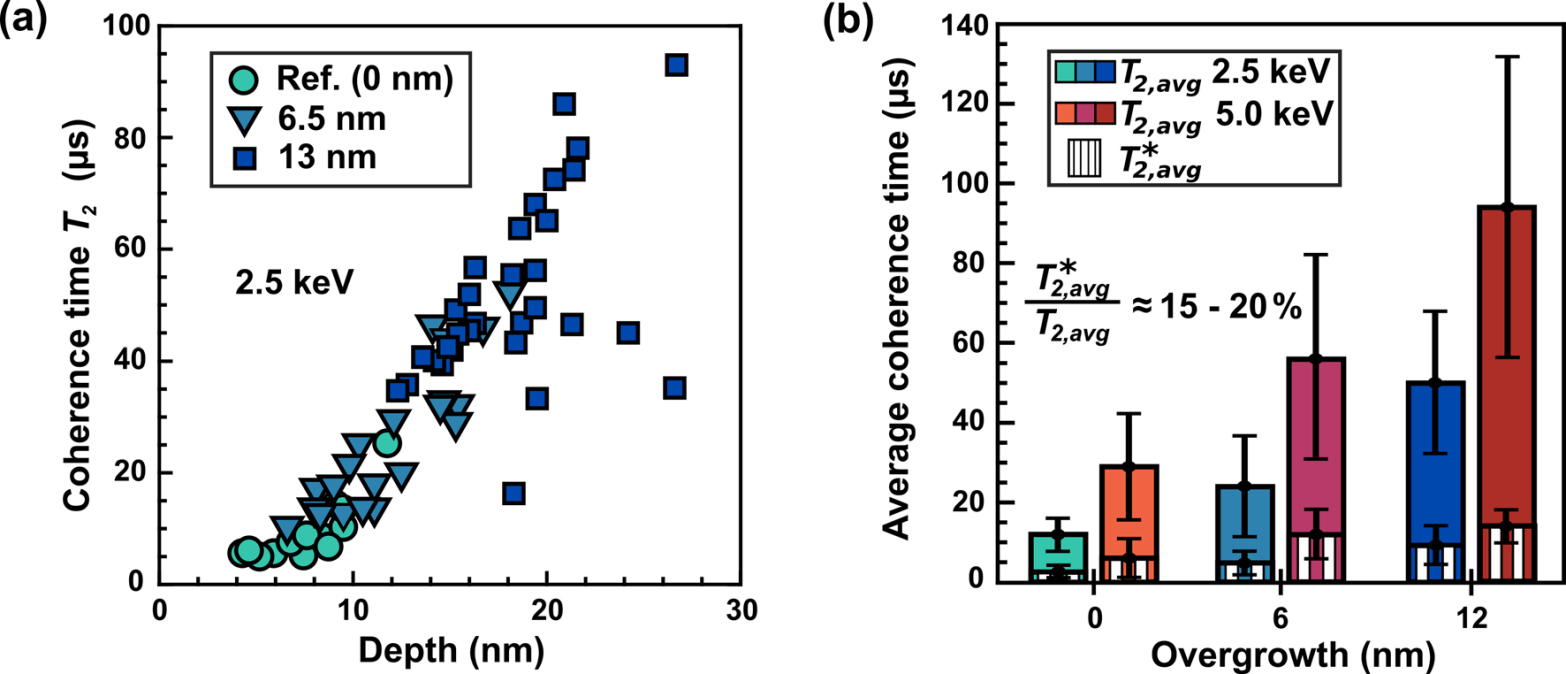
Enhancing the coherence time of single $\hbox {NV}^{-}$ centers by varying the thickness of the protective capping layer. (**a**) The scaling of $T_{2}$ (Hahn-echo) with depth for the reference and two capping-layer thicknesses, namely 6.5 nm and 13 nm. The values in the legend correspond to the layer thicknesses expected from the average growth rate in Fig. 2b. (**b**) Comparison of the average values for $T_{2,avg}$ and $T_{2,avg}^{*}$ and their evolution with the thickness of the overgrown layer for implantation energies of 2.5 keV and 5 keV.

As already reported in the literature, $T_{2}$ increases significantly with the depth of the $\hbox {NV}^{-}$ centers^29,31,49,50^ (Fig. 3(a)). Furthermore, for depth values larger than 8 nm in Fig. 3(a) the relationship between $T_{2}$ and $\hbox {NV}^{-}$-depth appears to follow a linear trend but at this stage we do not have an explanation for this behavior. Comparing the values for $T_{2}$ of the reference with the ones measured in the overgrown samples, overgrowth clearly enhances the coherence time for the majority of the $\hbox {NV}^{-}$ centers. As observed for the depth distribution in Fig. 2(a), we obtain a uniform prolongation of $T_{2}$ through overgrowth. As a consequence, the growth interface seems to play a minor role for the spin properties compared to the diamond surface.

In Fig. 3(b) we present the average values $T_{2,avg}$ and $T_{2,avg}^{*}$ for the samples studied in Fig. 3(a). Additionally, we show the data for a 5 keV implantation and the error bars in Fig. 3(b) correspond to the $1{\upsigma }$-standard deviation of all measured coherence times. By overgrowth, we enhance $T_{2,avg}$ significantly irrespective of the implantation energy. By burying $\hbox {NV}^{-}$ centers with a diamond layer of 13 nm thickness, we find a five-times longer $T_{2,avg}$ for 2.5 keV and three-times longer for 5 keV.

Likewise, the average $T_{2,avg}^{*}$ time rises as well and after overgrowth we observe very high values of around $20\,{\upmu }\hbox {s}$. It is interesting to note that $T_{2,avg}^{*}$ reaches between 15 and $20\,\%$ of the corresponding $T_{2,avg}$ before and after the overgrowth which can be interpreted as a non-changing spin environment for the $\hbox {NV}^{-}$ centers. So the indirect overgrowth procedure does not create additional spin baths, apart from the weakening influence of the surface, and indeed improves $T_{2}^{*}$ and $T_{2}$.

Note that although the $\hbox {NV}^{-}$ centers are buried below a nanometer-thick diamond layer, we still detect the nano-NMR signal from the $^{1}\hbox {H}$-spins in the immersion oil on the surface. Therefore, by controlled overgrowth on the nanometer scale we enhance significantly the coherence times while still preserving a sufficient sensitivity to nuclear spins at the surface.

After overgrowth and subsequent annealing (Fig. 1(b)), however, we either observe less $\hbox {NV}^{-}$ centers (medium and high dose) compared to a sample annealed directly after implantation or no $\hbox {NV}^{-}$ centers (low dose) at all. Note that the missing $\hbox {NV}^{-}$ centers are not present in their neutral charge state ($\hbox {NV}^{0}$). As we have already excluded etching during the CVD process (see above), a possible explanation is the passivation of NV centers by hydrogen forming NVH.

**Figure 4:**
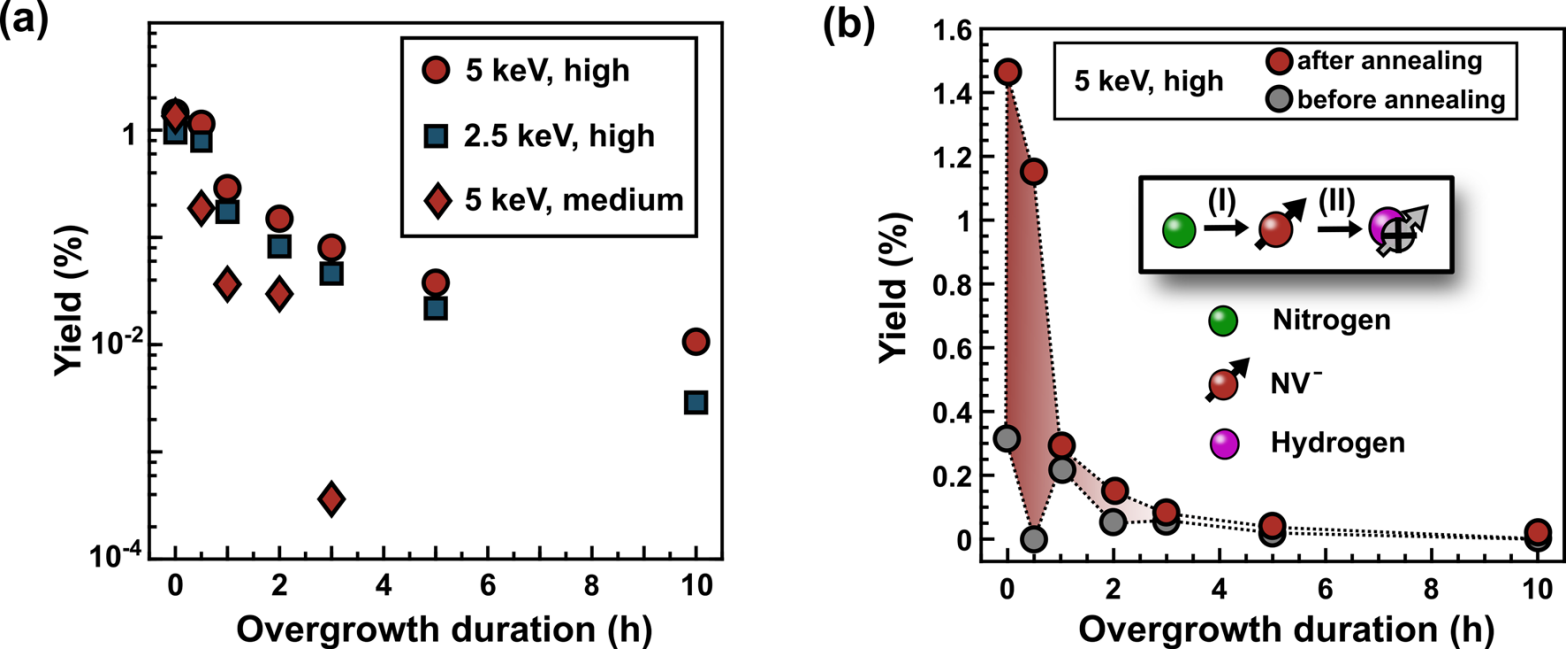
The loss of $\hbox {NV}^{-}$ centers caused by passivation during overgrowth. (**a**) The evolution of the yield over time in dependence of the implantation dose and energy. The yield is estimated by analyzing the $\hbox {NV}^{-}$ fluorescence profiles and comparing them with the one of a single $\hbox {NV}^{-}$ center. For details refer to C. Osterkamp et al.^40^. (**b**) Comparison of the yield directly after overgrowth with the one after annealing. The red shaded area is a measure for the nitrogen not converted into $\hbox {NV}^{-}$ centers during the CVD process. The schematic proposes a two-step mechanism for the passivation consisting of the formation of $\hbox {NV}^{-}$ centers (I) before hydrogen from the plasma can passivate them (II).

To study the influence of implantation energy and dose on the passivation kinetics, we estimate in Fig. 4(a) the number of remaining $\hbox {NV}^{-}$ centers after overgrowth for durations between zero and ten hours. For this, we calculate the average $\hbox {NV}^{-}$ density per confocal volume by analyzing the fluorescence profiles after annealing, similar to the technique presented C. Osterkamp et al.^40^. Finally, we determine the yield after overgrowth and annealing with respect to the implanted dose of nitrogen. The yield after annealing in Fig. 4(a) decreases rapidly with increasing overgrowth time regardless of the implantation energy or the dose. Increasing the implantation energy from 2.5 to $5\,\hbox {keV}$ for the high dose seems to have a minor influence on the speed of the passivation of the NV centers, as the decay of the respective curves in Fig. 4(a) looks similar. The slightly higher yield for $5\,\hbox {keV}$ (high dose) in Fig. 4(a) is related to the fact that higher implantation energies usually lead to enhanced $\hbox {NV}^{-}$ yields due to higher vacancy densities^51^. By contrast, keeping the implantation energy at $5\,\hbox {keV}$ but changing from the high to the medium dose, we observe a relatively fast passivation of the $\hbox {NV}^{-}$ centers within 3 h. Therefore, the amount of implanted nitrogen into the diamond affects the speed of passivation drastically and in our experiments the $\hbox {NV}^{-}$ centers related to the high dose implantation turn out to be more stable towards passivation than the lower dose ones.

In Fig. 4(b) the yield of the high dose in the overgrown samples is compared before and after the annealing step. The difference between the two curves is shaded in red and corresponds to the amount of nitrogen that is converted into $\hbox {NV}^{-}$ centers through annealing and not due to the temperature of $900\,^{\circ }\hbox {C}$ during the CVD process. In the early stages of the overgrowth process, hardly $\hbox {NV}^{-}$ centers seem to form from the implanted nitrogen and mobile vacancies. As a consequence, the majority of $\hbox {NV}^{-}$ is generated by annealing only (red area in Fig. 4(b)). For overgrowth processes longer than 30 min, the added yield by annealing gets marginal and we observe most of the $\hbox {NV}^{-}$ centers already directly after overgrowth. Once $\hbox {NV}^{-}$ centers are formed, they can get passivated (cf. direct overgrowth). Therefore, to explain the losses in $\hbox {NV}^{-}$ centers also during the indirect overgrowth, we propose a two-step mechanism where the formation of $\hbox {NV}^{-}$ centers (step I) is a prerequisite for their subsequent passivation (step II), as illustrated in the schematic inset in Fig. 4(b). According to the relatively large red area in Fig. 4(b), step I seems to be significantly slower than step II limiting the losses by passivation within the first 30 min of overgrowth. The drop of the overall yield in this time equals the amount of $\hbox {NV}^{-}$ centers already present in the reference before annealing. These initial centers form during heating the sample holder to approx. $\hbox {700}\,^{\circ }\hbox {C}$ prior to growth and get probably quickly passivated as seen for the direct overgrowth.

Between 30 min and one hour, step I appears to accelerate and, as a result, the yield drops by one order of magnitude due to relatively fast passivation in step II. After one hour, however, step II seems to slow down as we observe NV centers directly after overgrowth without any further annealing in Fig. 4(b). Moreover, the slower passivation is also reflected in a marked flattening of the yield curves in case of the high dose in Fig. 4(a). On the contrary, for the medium dose we find no signs for a change in the passivation rate and based on the quick drop of the yield for the medium dose in Fig. 4(a) we assume that both step I and II are faster than in case of the high dose.

Since in our case the implanted nitrogen in the diamond seems to affect the kinetics of the passivation, we suspect that the implanted nitrogen hinders the diffusion of hydrogen in diamond. From ab-initio calculations it is expected that the positively-charged hydrogen exhibits the highest mobility in diamond^52^. An electron from a donor, e.g. nitrogen^53^, could change the hydrogen’s charge state from positive to neutral or negative and, therefore, slow down its diffusion^52^. As a consequence, in the layer implanted with the medium dose, the charging of hydrogen would occur less frequently than in case of the high dose and, therefore, lead in the end to a higher average mobility of hydrogen and a faster passivation.

To our knowledge charging of diffusing hydrogen by nitrogen-dopants in diamond has not been reported, yet, but dopants like boron^54^ or sulfur^55^ have already been associated with charging of vacancies in diamond. Hence, we believe that the nitrogen-dependent mobility of hydrogen in diamond might be an explanation for the strong influence of the implantation dose on the success of the overgrowth of NV centers.

## Conclusion

In summary, we have shown that indirect overgrowth as a synergy of nitrogen ion implantation and CVD-diamond growth is a powerful method to fabricate shallow $\hbox {NV}^{-}$ center. Due to the usage of smaller implantation energies, the observed depth distributions are more narrow than the ones expected from higher implantation energies necessary to create NV centers in comparable depths by implantation only.

Furthermore, we verify that the enhanced coherence times after overgrowth are indeed related to the increased average depth of the $\hbox {NV}^{-}$ centers. By overgrowing a capping layer of 13 nm on top of implanted nitrogen we achieve coherence times up to $100\,{\upmu }\hbox {s}$ for $\hbox {T}_{2}$ and remarkable $20\,{\upmu }\hbox {s}$ for $T_{2}^{*}$ for single $\hbox {NV}^{-}$ centers without losing the sensitivity to protons in the immersion oil at the surface. We attribute the losses in $\hbox {NV}^{-}$ centers during overgrowth to the passivation by hydrogen as we can exclude severe etching of diamond. The passivation rate seems to decrease significantly with increasing implantation dose. This dose can be tuned to stabilize the $\hbox {NV}^{-}$ centers during overgrowth without compromising their spin properties. Additionally, by employing indirect overgrowth we mitigate passivation as long as no $\hbox {NV}^{-}$ centers form during growth.

As a consequence, indirect overgrowth is a promising tool for the controlled and repeatable creation of stable color centers in diamond. The synergetic combination of low energy ion implantation and nanometer-precise CVD diamond growth in the presented manner allows stable creation of depth-confined NV-qubits with excellent external spin sensing capabilities. In addition, a more detailed study of the passivation effect can be used to investigate the dynamics of NV formation and deactivation. Gaining insight into these atomic processes inside the crystal is an essential task towards the goal of improved defect generation for the realization of a diamond quantum sensor with enhanced sensitivity.

## Methods

### Diamond substrates

We employ commercial electronic-grade (100)-diamond substrates from Element Six and clean them before growth and confocal microscopy analysis in an ultrasonic bath with acetone, isopropanol, and deionized water. Subsequently, we clean the samples with a mixture (1:1:1 volume ratio) of nitric ($65\,\%$), sulphuric ($97\,\%$), and perchloric acid ($70\,\%$) in a microwave reactor system (MWT AG, type ETHOS.Lab) at $200\,^{\circ }\hbox {C}$ for 30 min.

### CVD growth

A detailed description of the home-built CVD reactor has been published by Silva et al.^56^ and in a work of Osterkamp et al.^40^. The commercial substrates are overgrown with an ultrapure $^{12}\hbox {C}$-diamond layer with a thickness of roughly 150 nm using $99.999\,\%$ enriched $^{12}\hbox {CH}_{4}$ gas (Cambridge Isotope Laboratories) at a concentration of $0.2\,\%$ with respect to hydrogen. The latter is applied at a flow rate of 600 sccm and a working pressure of $22.5\,\hbox {mbar}$ while the microwave power is set to $1.2\,\hbox {kW}$. The buffer layer serves as a starting point for our experiments. For the indirect/direct overgrowth the $^{12}\hbox {C}$ methane concentration is reduced to $0.05\,\%$ with respect to hydrogen. Before growth the sample holder is heated to $700\,^{\circ }\hbox {C}$ with a graphite substrate heater. Then the sample gets exposed to a hydrogen plasma for five minutes before the methane is injected and the capping layer is grown at $900\,^{\circ }\hbox {C}$. The temperature in the CVD system is measured by an infra-red pyrometer (Optris, type CTlaser 2 MH, wavelength $1.6\,{\upmu }\hbox {m}$). The employed process gases are purified with a palladium filter (Johnson Matthey, Hydrogen Purifier HP-25) and a getter-filter for methane (MonoTorr, PS4-MT3-531).

### Ion implantation and UHV annealing

Ion implantation and annealing apparatuses used for $\hbox {NV}^{-}$ center generation are described in detail in a previously published study on shallow implanted silicon vacancy centers^57^. The home-built low energy ion implanter (featuring ion source IQE12/38 from Specs) is equipped with a Wien mass filter to select the desired ion species and utilizes an Einzel lens to focus the ion beam to spot diameters of approximately $50\,{\upmu }\hbox {m}$. As the nitrogen source we use $98\,\%$-enriched $^{15}\hbox {N}_{2}$ gas (Sigma-Aldrich). The samples are annealed in a UHV oven at $1000\,^{\circ }\hbox {C}$ for 3 h ensuring process pressures below $1\times 10^{-7}\,\hbox {mbar}$ which prevents severe surface graphitization.

### Confocal microscopy and spin properties

In our home-built scanning confocal microscopy setup we employ for the excitation a pulsed $519\,\hbox {nm}$ laser (Toptica Photonics, type iBeam-smart-515-S) which is focused onto the diamond with an oil immersion objective (Olympus UPlanSApo, 60x/1.35 NA). The fluorescence of the $\hbox {NV}^{-}$ centers is collected by the same objective and gets detected by an avalanche photo diode with a $638\,\hbox {nm}$ longpass filter. The control of the electronic spin is achieved by applying microwaves from a continuous wave MW generator (Rohde und Schwarz, SMIQ04B) or microwave pulses from an arbitrary waveform generator (Tektronix, AWG700001A) through a copper wire. The magnetic field alignment is performed with a ferromagnet mounted on a 3D-rotatory stage. The pulsed ODMR measurements are conducted at a magnetic field of 60 G along the $\hbox {NV}^{-}$-symmetry axis, while for the Ramsey, Hahn-echo, and XY8 pulse sequences a field of 500 G is applied. The microwave power is adjusted to obtain Rabi periods around 30 ns. For the control of the experiments, we use the software package qudi^58^.

## Data Availability

The datasets generated during and/or analysed during the current study are available from the corresponding authors on reasonable request.
